# Influence of Turning Intervals on Microbial Succession and Volatile Aroma Development During On-Farm Cocoa Bean Fermentation

**DOI:** 10.3390/foods15213814

**Published:** 2026-10-25

**Authors:** Kridsada Unban, Tabkrich Khumsap, Apinun Kanpiengjai, Kalidas Shetty, Chartchai Khanongnuch

**Affiliations:** 1 Division of Food Science and Technology, School of Agro-Industry, Faculty of Agro-Industry, Chiang Mai University, Mueang, Chiang Mai 50100, Thailand; tabkrich.khumsap@cmu.ac.th; 2 Division of Biochemistry and Biochemical Innovation, Department of Chemistry, Faculty of Science, Chiang Mai University, Chiang Mai 50200, Thailand; apinun.k@cmu.ac.th; 3 Department of Plant Pathology, Microbiology and Biotechnology, North Dakota State University, Fargo, ND 58102, USA; kali-das.shetty@ndsu.edu; 4 Center of Excellence in Agricultural Innovation for Graduate Entrepreneur, Maejo University, Chiang Mai 50290, Thailand; chartchai_knn@mju.ac.th

**Keywords:** cocoa fermentation, microbial dynamics, turning interval, bacterial community, yeast community, agricultural productivity, sustainable agriculture

## Abstract

The present study examined the effects of microbial communities and their associated physicochemical characteristics during on-farm cocoa fermentation in Chiang Mai, Northern Thailand, evaluated at a 60-kg wooden box scale. Two fermentation processes, 24- and 48-h interval turning processes, were comparatively evaluated. Microbial populations were monitored using both culture-based enumeration and culture-independent molecular techniques. The results showed that lactic acid bacteria and acetic acid bacteria were the predominant microbial populations throughout fermentation. Multivariate analysis found that fermentation time significantly shifted the community from yeast-based communities (*Saccharomyces* and *Hanseniaspora*) and bacterial (*Limosilactobacillus fermentum)* towards a late-stage community dominated by acid-tolerant *Acetobacter pasteurianus*. Moreover, the turning interval strongly influenced the microbial community, with a 24-h interval favoring *Saccharomyces* and *Komagataeibacter*, whereas a longer 48-h interval selectively promoted *Candida*, *Meyerozyma*, and *Acinetobacter*. Physicochemical properties also showed that total acidity increased and pH decreased in both processes, while reducing sugars declined over time. In the 24-h turning process (T24), reducing sugars decreased from 64.80 mg/g at 0 h to 3.73 mg/g at 144 h. Similarly, in the 48-h turning process (T48), reducing sugars decreased from 64.29 mg/g at 0 h to 3.21 mg/g at 144 h. Pectinase activity was detected as the main enzyme activity in both processes. In addition, the T24 process showed that *Saccharomyces*, *Limosilactobacillus*, and *Lentilactobacillus* were positively correlated with key fruity/sweet esters, whereas *Lentilactobacillus* and *Acinetobacter* were correlated with volatile markers linked to off-flavor notes (butanoic acid and dimethyl disulfide) in the T48 process. Overall, more frequent turning clearly promotes more volatile compounds associated with desirable aroma profiles, and standardizing turning schedules could help improve batch-to-batch consistency for aroma development and reduce the risk of undesirable flavor formation during scale-up.

## 1. Introduction

Cocoa (*Theobroma cacao* L.) is the main raw material used in chocolate production. To achieve chocolate’s distinctive sensory qualities, raw cocoa beans must undergo several processing steps, including fermentation, drying, and roasting [1,2]. Among these stages, fermentation is particularly critical to final product quality. This biological process converts sugars and acids while reducing unwanted bitterness and astringency [2,3]. Moreover, fermentation generates the essential flavor and aroma precursors that are fully realized during roasting, ultimately defining chocolate’s distinct sensory profile [4,5].

Cocoa bean fermentation is a spontaneous microbial process that occurs on the farm through traditional practices. Fermented beans are then processed at downstream facilities in the food-processing chain [6]. This spontaneous fermentation exhibits a microbial succession of yeasts, lactic acid bacteria (LAB), and acetic acid bacteria (AAB) [7]. Their metabolic activity degrades cocoa pulp into end products, transforming the seeds into flavor-enriched stable beans optimized for subsequent drying, roasting, and chocolate production. Spontaneous fermentation is a complex process driven by numerous fungi and bacteria in specific growing and processing environments [8]. The cocoa pods are naturally inoculated with various fungi and bacteria from the local ecosystem at harvest and are subsequently influenced by this microbiota during fermentation and drying. The fermentation drives environmental changes that sequentially select for microbial communities adapted to each stage of the process. Interactions among these microorganisms play an important role in influencing the final flavor and aroma of cocoa [9]. Yeasts are typically among the first microorganisms found in cocoa fermentation. They use the pulp’s high sugar content and convert it into ethanol, carbon dioxide, and volatile compounds, thereby increasing the overall temperature [4]. Both culture-dependent and culture-independent studies identified *Saccharomyces*, *Pichia*, and *Hanseniaspora* as the predominant fungal genera [4,9,10]. Each yeast species has a distinct metabolic profile, expressing different enzymes and pathways that produce a range of compounds in varying amounts. LAB generally represents the second main microbial population during cocoa fermentation. LAB metabolites such as lactic acid, glycerol, mannitol, and volatile compounds are important for chocolate flavor development [9]. Common LAB strains, including the genera *Lactobacillus*, *Leuconostoc*, and *Pediococcus*, have been reported in cocoa bean fermentation [7,8,11,12]. The final microbial group is AAB, mainly from the genera *Acetobacter* and *Gluconobacter*, which utilize ethanol and convert it into acetic acid as a metabolic end product [7,11,13].

Cocoa cultivation in Thailand has expanded in recent years, increasing the importance of post-harvest practices that determine bean quality and market value. Local cooperatives and smallholder farmers increasingly recognize fermentation as a key process that influences the physicochemical properties, sensory quality, and commercial competitiveness of cocoa beans [14]. Properly fermented beans with desirable fine-flavor characteristics and potentially beneficial bioactive compounds may command substantially higher prices than poorly fermented or unfermented beans. During cocoa fermentation, turning is a commonly studied processing step that influences fermentation conditions and bean outcomes, with research showing effects on sensory profiles, aroma composition, acidity, microbial dynamics, and overall bean quality [15,16,17,18]. Studies from cocoa-producing regions such as Ivory Coast, Ghana, and other major producing areas have examined different turning intervals, including 24 h and 48 h schedules, and reported that the turning regime can affect fermentation duration, organic acid profiles, and final bean quality [15,16,18,19,20]. However, turning efficiency depends on batch scale, container type, and regional climate conditions. Consequently, improving fermentation practices may increase smallholder incomes while supporting the sustainable development and competitiveness of Thailand’s domestic craft chocolate sector [14,21]. Cocoa has become a promising economic crop in Thailand, underscoring the need for fermentation processes that are controlled, reproducible, and adaptable to local production conditions. Obtaining consistent fermentation is not only a scientific and technological challenge but also an economic imperative for enhancing the quality, value, and market competitiveness of Thai cocoa within the regional chocolate value chain [22]. However, smallholder cocoa farmers often face limitations in labor availability and time. Frequent turning of cocoa during fermentation can increase labor demands and may not be feasible for small-scale producers [23,24]. Therefore, identifying practical turning intervals that support consistent fermentation while reducing labor requirements is important for improving the feasibility of on-farm cocoa fermentation. Nevertheless, scientific evidence regarding the temporal dynamics of microbial communities and their relationship to quality development during on-farm cocoa fermentation in Thailand remains limited.

To clarify the influence of turning interval times in local small-scale producers, this study investigated the microbial community dynamics during cocoa fermentation by comparing two turning intervals. Culture-dependent methods and culture-independent high-throughput sequencing were employed to characterize temporal changes in bacterial and fungal communities throughout the fermentation process. In addition, physicochemical parameters were monitored to examine their association with microbial succession under the two fermentation processes.

## 2. Materials and Methods

### 2.1. Cocoa Fermentation Process and Sample Collection

**Figure 1:**
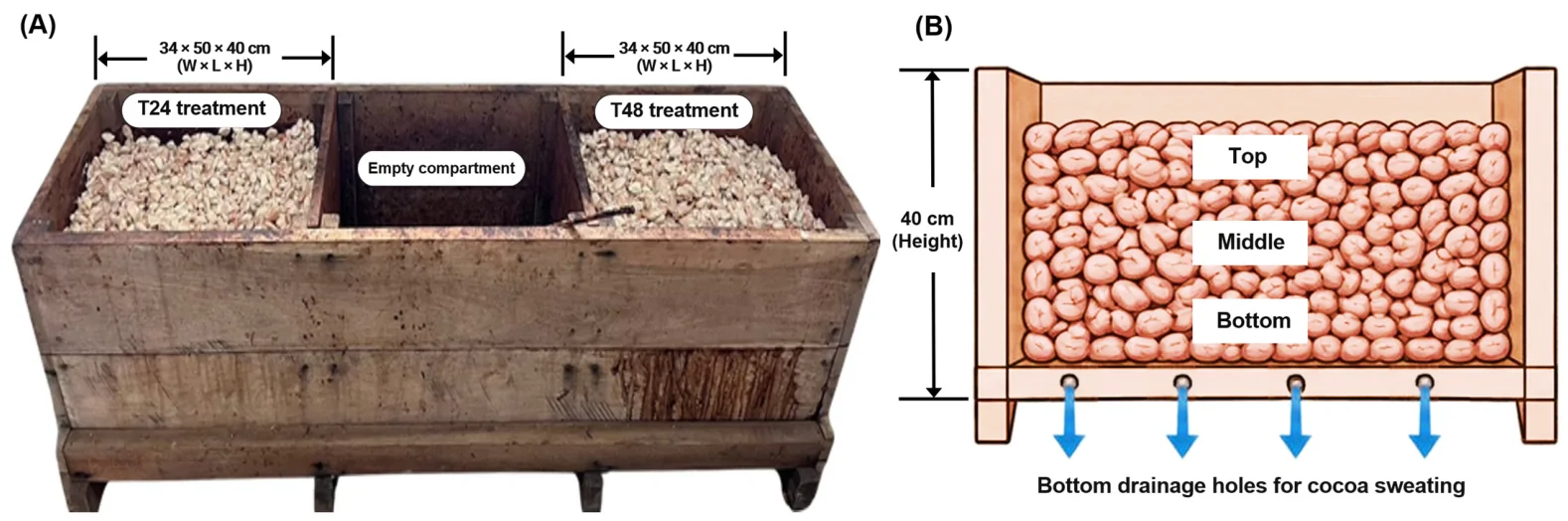
Schematic diagram of the wooden cocoa fermentation box used in this study, shown in top view (**A**) and cross-sectional side view (**B**). Samples from the top, middle, and bottom positions are pooled as one composite sample.

Fully ripe cocoa pods (*Theobroma cacao* L.) of the Chumphon 1 cultivar (the PA7 and NA32 varieties of Trinidad hybrid) were sourced from a local chocolate-producing company in Chiang Mai, Thailand. The pods were cut horizontally with a sharp knife without damaging the beans. Visibly spoiled or germinated pods were discarded, and only high-quality pods were selected for further use. The on-farm fermentation was conducted using a wooden box (34 × 50 × 40 cm) divided by solid wooden partitions with a capacity of 60 kg per treatment (Figure 1), which operated simultaneously during the same harvest period. The wooden fermentation boxes were equipped with drainage holes at the bottom to allow the liquid released during fermentation to drain out. The wooden box was covered with a thin white cloth. The fermentation process lasted 6 days (144 h) and consisted of two experimental fermentations. In the first process (T24), the beans were turned manually every 24 h; in the second process (T48), the beans were turned every 48 h. Sampling was conducted every 24 h (0, 24, 48, 72, 96, 120, and 144 h) over the 6 days. For the T48 treatment on non-turning days, samples from the lower region were collected carefully by gently moving aside a small portion of the fermenting mass. At each sampling time point, approximately 200 g of fermented cocoa beans were randomly collected from the top, middle, and bottom regions of the box and pooled to obtain a representative composite sample for fermentation monitoring and immediate microbiological analysis. The remaining cocoa bean samples were placed in polyethylene bags and stored at −20 °C for subsequent physicochemical analysis.

### 2.2. Enumeration of Microorganisms by Culture-Dependent Methods

For microbial enumeration, 10 g of cocoa beans were aseptically transferred into a Duran flask containing 90 mL of sterile 0.85% (*w*/*v*) NaCl solution, and the mixture was manually agitated for 5 min to obtain a homogeneous suspension of cocoa bean pulp. The diluted samples from 10^−2^ to 10^−8^ were spread on potato dextrose agar (PDA) (HiMedia, Nashik, India) acidified by adding 18 mL of sterile 10% (*w*/*v*) tartaric acid solution per 1000 mL of sterilized medium; de Man, Rogosa, and Sharpe (MRS) agar (HiMedia, Nashik, India) supplemented with 0.015% (*w*/*v*) bromocresol purple; acetic acid bacteria-selective agar (ABS) (HiMedia, Nashik, India) supplemented with 0.02% (*w*/*v*) bromophenol blue; and MacConkey agar plates for enumeration of yeast, LAB, AAB, and coliform bacteria, respectively. The remaining sample solution was heated at 80 °C for 12 min, and the proper dilutions were spread on NA for spore-forming bacteria. The sample preparation, dilutions, and plating methods were performed according to the methodology described by Unban et al. [25]. The inoculated agar plates were incubated at 30 °C for 3 days, after which microbial populations were enumerated and expressed as colony-forming units per gram of sample (CFU/g).

### 2.3. Culture-Independent Analysis of Microbes

#### 2.3.1. Extraction of Genomic DNA

Total genomic DNA from cocoa bean pulp was extracted as described previously with some modification [26]. Briefly, 20 g of cocoa bean was mixed with 10 mL of saline solution. The cocoa bean was removed manually, and the remaining mixture was centrifuged at 1000 rpm for 5 min at 4 °C to remove the plant material by decantation. The supernatant was then filtered through a 20-μm pore-size filter and centrifuged at 10,000 rpm for 10 min at 4 °C to obtain the presumed cell pellets. The resuspended pellets of each sample were extracted with the DNeasy^®^ PowerSoil^®^ Kit (Qiagen, Valencia, CA, USA). The concentration and purity of the extracted DNA were evaluated using a NanoDrop 2000 spectrophotometer (Thermo Fisher Scientific, Inc., Waltham, MA, USA). DNA samples were considered suitable for downstream analysis when they met the following criteria: a concentration of at least 50 ng/µL, a minimum total volume of 20 µL, and an A260/A280 ratio between 1.8 and 2.0. Genomic DNA was stored at −20 °C until further processing. Qualified samples were then used to amplify the bacterial 16S rRNA gene and fungal internal transcribed spacer (ITS) region, followed by high-throughput sequencing and bioinformatic analysis.

#### 2.3.2. Amplicon Sequencing of 16S rRNA Gene

The V3-V4 region of the bacterial 16S rRNA gene was amplified by PCR using universal primers 338f (5′-ACTCCTACGGGAGGCAGCA-3′) and 806r (5′-GGACTACHVGGGTWTCTAAT-3′) in the V3-V4 region of the 16S rRNA gene for bacteria, with a specific barcode at the 5′ end. The PCR reactions were carried out with Phusion High-Fidelity PCR Master Mix (New England Biolabs, Beverly, MA, USA).

Thermal cycling was performed under the following conditions: an initial denaturation step at 95 °C for 5 min; 25 cycles of denaturation at 95 °C for 30 s, annealing at 56 °C for 30 s, and extension at 72 °C for 40 s; followed by a final extension at 72 °C for 10 min. The resulting PCR amplicons were verified by electrophoresis on a 2% (*w*/*v*) agarose gel. Amplicons were subsequently pooled and purified using the Qiagen Gel Extraction Kit (Qiagen, Germany). Qualified libraries were sequenced on an Illumina NovaSeq 6000 platform (Illumina, San Diego, CA, USA) by Beijing Biomarker Technologies Co., Ltd. (Beijing, China). Raw sequencing reads were initially quality-filtered using Trimmomatic v0.33. Primer sequences were identified and removed using Cutadapt v1.9.1 to obtain high-quality, primer-free reads. Denoising and chimera removal were then conducted using the DADA2 algorithm implemented in R, resulting in high-quality non-chimeric reads for subsequent analysis.

#### 2.3.3. Amplicon Sequencing of the ITS Region

The ITS1 rRNA gene of yeast was amplified using primers ITS-1 (5′-CTTGGTCATTTAGAGGAAGTAA-3′) and ITS-2 (5′-GCTGCGTTCTTCATCGATGC-3′) with a specific barcode at the 5′ end. The PCR reactions were carried out with Phusion High-Fidelity PCR Master Mix (New England Biolabs, Beverly, MA, USA). Thermal cycling was performed under the following conditions: initial denaturation at 95 °C for 5 min; 25 cycles of denaturation at 95 °C for 30 s, annealing at 56 °C for 30 s, and extension at 72 °C for 40 s; followed by a final extension at 72 °C for 10 min. PCR amplicons were evaluated by electrophoresis on a 2% (*w*/*v*) agarose gel. The amplicons were subsequently pooled and purified using a Qiagen Gel Extraction Kit (Qiagen, Germany). Qualified libraries were sequenced using an Illumina NovaSeq 6000 platform (Illumina, San Diego, CA, USA) by Beijing Biomarker Technologies Co., Ltd. (Beijing, China).

#### 2.3.4. Bioinformatics Analysis

Raw sequencing reads were quality-filtered using Trimmomatic v0.33, and primer sequences were removed using Cutadapt v1.9.1. The resulting reads were processed using the DADA2 pipeline in R, including filtering, dereplication, error-model learning, denoising, paired-end read merging, and chimera removal. This procedure generated amplicon sequence variants (ASVs). Taxonomic assignment of representative ASV sequences was performed in QIIME 2 (version 2020.6) using the UNITE database for fungi and the SILVA 138 database for bacteria. Alpha diversity was assessed using the Shannon diversity index calculated with a base-2 logarithm (log_2_). Redundancy analysis (RDA) and canonical correspondence analysis (CCA) were performed to evaluate the relationships between bacterial community composition and environmental factors. All ordination analyses were conducted in R using the vegan package (version 2.3).

### 2.4. Monitoring of Temperature, pH, and Total Acidity During Cocoa Fermentation

Temperature measurements were conducted using a digital pen thermometer with a range of 0–110 °C. The thermometer was dipped into the fermenting cocoa bean samples, and readings were taken every 24 h. Temperature was measured at the center of the fermentation box, approximately midway through the cocoa bean mass, at each turning event. The pH of the cocoa pulp was measured using a Starter 2100 pH meter (OHAUS, Pine Brook, NJ, USA). Total titratable acidity was determined by sodium hydroxide (NaOH) titration according to the methods of the Association of Official Analytical Chemists [27]. Briefly, 10 g of cocoa bean sample was suspended in 100 mL of sterile distilled water and homogenized for 10 min using a Masticator homogenizer (IUL Instruments, Barcelona, Spain). The homogenate was centrifuged at 10,000 rpm and 4 °C for 5 min to separate the supernatant. The resulting supernatant was then used to determine total titratable acidity by NaOH titration.

### 2.5. Determination of Reducing Sugar and Enzyme Activity

Reducing sugar content in cocoa bean samples was determined using the 3,5-dinitrosalicylic acid (DNS) colorimetric method [28]. Briefly, 10 g of cocoa bean sample was suspended in 100 mL of distilled water and centrifuged at 10,000 rpm for 10 min. The resulting supernatant was diluted appropriately before analysis. A 500 µL aliquot of the diluted sample was mixed with an equal volume of DNS reagent in a test tube and heated at 100 °C for 10 min. After cooling to room temperature, 4 mL of distilled water was added to the reaction mixture. Absorbance was measured at 540 nm using a spectrophotometer, and reducing sugar concentration was calculated from a glucose standard calibration curve.

The activities of pectinase, amylase, carboxymethyl cellulase, mannanase, and xylanase were determined using the 3,5-dinitrosalicylic acid (DNS) method, based on the quantification of reducing sugars released from their respective substrates: pectin (Wako Pure Chemical, Tokyo, Japan), soluble starch (KemAus, Cherrybrook, Australia), carboxymethyl cellulose (CMC) (Nacalai Tesque, Inc., Tokyo, Japan), locust bean gum (Sigma-Aldrich, St. Louis, MO, USA), and beech wood xylan (Megazyme, Wicklow, Ireland). The assay was performed according to a method modified from Unban et al. [29]. The extracellular enzymes were extracted by homogenizing 10 g of cocoa beans in 100 mL of ice-cold 50 mM sodium phosphate buffer (pH 7.0) for 10 min, followed by centrifugation at 10,000× *g* for 10 min at 4 °C. The clarified supernatant was used as the crude enzyme extract. The diluted crude enzyme volume of 0.125 mL was mixed with 0.125 mL of the 0.5% (*w*/*v*) respective substrate solution prepared in 50 mM sodium phosphate buffer (pH 7.0). The reaction mixture was incubated at 37 °C for 15 min, after which 0.250 mL of DNS reagent was added. The mixture was then boiled for 10 min to terminate the enzymatic reaction. Subsequently, 2 mL of distilled water was added, and absorbance was measured at 540 nm. Enzyme activities were expressed as units per milliliter (U/mL), where one unit was defined as the amount of enzyme required to release 1 µmol of reducing sugars per minute under the specified assay conditions [30].

### 2.6. GC E-Nose Analysis for the Volatiles

The volatile profiles of the cocoa nib samples were analyzed using a Heracles NEO electronic nose system (Alpha M.O.S., Toulouse, France) equipped with an automated headspace sampler. Volatile organic compounds (VOCs) were separated using two capillary columns, MXT-5 and MXT-1701, and detected using two flame ionization detectors (FIDs). Briefly, 2 g of ground cocoa nib sample was placed in a 20 mL headspace vial and sealed with a magnetic polytetrafluoroethylene (PTFE)/silicone cap. Samples were incubated at 50 °C for 20 min, after which 5 mL of headspace gas was injected into the Heracles gas chromatograph. A flushing time of 90 s was applied between injections. Helium was used as a carrier gas at a flow rate of 30 mL/min. VOCs were trapped at 40 °C. The oven temperature was initially maintained at 50 °C for 5 s, increased to 80 °C at a rate of 1 °C/s, and then increased to 250 °C at 3 °C/s, with a final holding time of 21 s. Both FIDs were maintained at 260 °C. Chromatographic peaks were converted into individual variables based on peak areas and their corresponding Kovats retention indices using AlphaSoft software, version 17.0 (Alpha M.O.S., Toulouse, France). Putative compound identification was performed by comparison with the AroChemBase database, version 2021 (Alpha M.O.S. Corporation, Toulouse, France). All samples were analyzed in triplicate.

### 2.7. Data Analysis and Statistics

All samples were analyzed in triplicate, and the results were calculated as the mean ± standard deviation (SD). One-way analysis of variance (ANOVA) was used to test for significant differences between means at the *p* < 0.05 level using SPSS statistical software, version 17.0 (SPSS Inc., Chicago, IL, USA). GraphPad Prism version 9.4.0 (GraphPad Software Inc., San Diego, CA, USA) was used to perform Pearson correlation analysis and to visualize the graphs.

## 3. Results

### 3.1. Microbial Changes During Cocoa Fermentation

**Figure 2:**
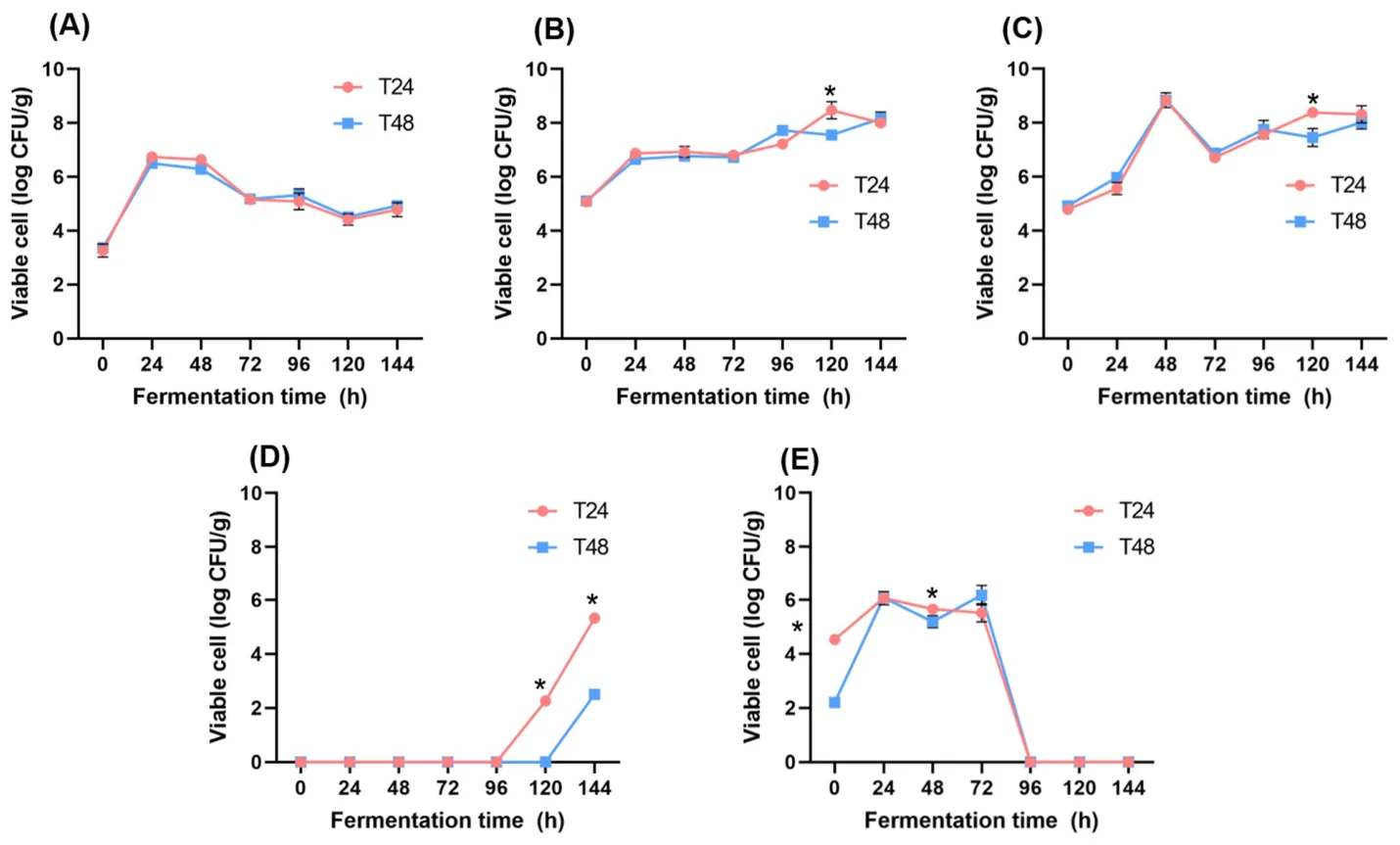
Viable microbial cell counts of yeast (**A**), acetic acid bacteria (**B**), lactic acid bacteria (**C**), spore-forming bacteria (**D**), and coliform bacteria (**E**) during the cocoa fermentation processes. Data are presented as the mean  ±  SD. The asterisk represents a significant difference between T24 and T48 at corresponding time points (*p* < 0.05).

The results of the microbial analysis during cocoa fermentation are presented in Figure 2. Viable microbial populations in both turning fermentation processes increased after 24 h, remained relatively stable through 72 h at approximately 6.5 log CFU/g, and then increased again to around 8.0 log CFU/g by 144 h. This trend aligns with established patterns of microbial succession during cocoa bean fermentation [31,32]. LAB dominated the early fermentation period (0–48 h), rising rapidly to a peak of about 9.0 log CFU/g after 48 h. Thereafter, LAB populations stabilized and declined slightly toward the final stages of fermentation in both processes, reaching approximately 8.5 log CFU/g, which corresponds to their metabolic activity during the initial anaerobic pulp degradation phase [31,33]. Yeast populations in both fermentation processes were similar, increasing quickly during the initial 24–48 h to reach a peak of around 6.5 log CFU/g, then gradually decreasing to approximately 5.0 log CFU/g by 144 h, reflecting their primary roles in ethanol production and pectin hydrolysis before microbial succession favored other groups [8]. Spore-forming bacteria were not detected during the initial 96 h in ×either fermentation process but became detectable later, at 120 h and 144 h for T24 and T48, respectively. Aerobic, spore-forming bacilli may proliferate during the later stages of fermentation, when temperatures increase, and conditions become less acidic and more aerobic [34]. Under fermentative conditions, aerobic spore-forming bacteria produce a range of chemical compounds that may contribute to the acidity and off-flavors of fermented cocoa beans [8]. In contrast, coliform bacteria appeared during the early fermentation phase (0–72 h) but were undetectable after 96 h, suggesting that coliforms represent early-stage environmental contaminants and were then reduced by increasing acidity.

### 3.2. Cluster Analysis of Microbial Populations of Fungi

Sequencing read processing results and alpha diversity indices for the ITS region are summarized in Tables S1 and S2, respectively. The Shannon diversity index was calculated to assess alpha diversity within the microbial communities during cocoa fermentation. As shown in Table 1, diversity varied across fermentation time points. For bacterial diversity, high diversity was observed during the early period of fermentation and then decreased markedly after 72 h of fermentation. In contrast, fungal diversity was high at the beginning of fermentation, decreased during the intermediate stages, and increased again after 72 h until the end of fermentation.

**Figure 3:**
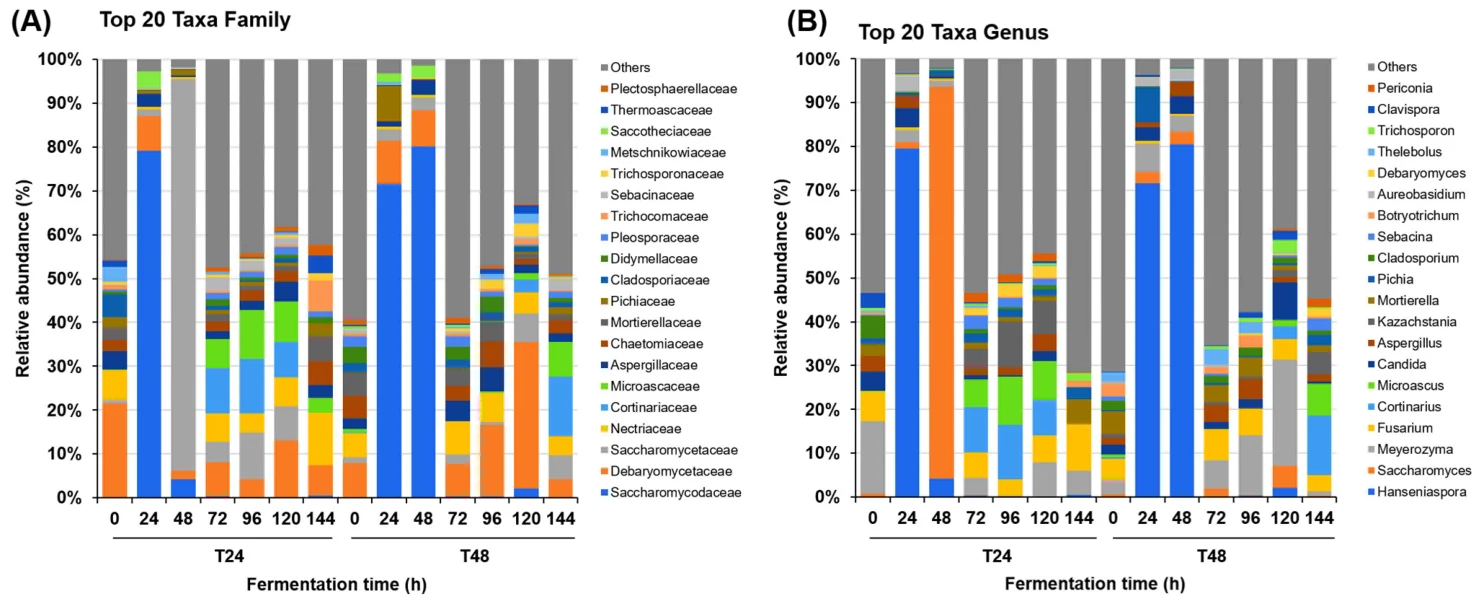
Fungal population groups at the family (**A**) and genus (**B**) levels during the cocoa fermentation processes.

Cluster analysis of fungal populations during cocoa fermentation representing the 20 most abundant fungal families and genera is presented in Figure 3. In the 24-h turning process (T24), *Hanseniaspora* yeasts, particularly *H. pseudoguilliermondii*, dominated at the beginning of the fermentation process, driving initial ethanol production, before shifting to *Saccharomyces cerevisiae* as the primary group by 48 h, followed by *Meyerozyma*, *Candida*, and *Cortinarius* in mid-fermentation (T24 at 72 and 96 h). *Fusarium circinatum* and *Microascus brevicaulis* increased in the late stage. In contrast, *H. pseudoguilliermondii* and *Meyerozyma caribbica* dominated the early stage of the T48 process (24 to 48 h), while *Saccharomyces* declined. *Meyerozyma caribbica* increased during late fermentation. These dynamics highlight the importance of frequent turning in promoting orderly yeast succession and the development of desirable flavor precursors. In contrast, infrequent turning may lead to a more dispersed fungal community.

**Figure 4:**
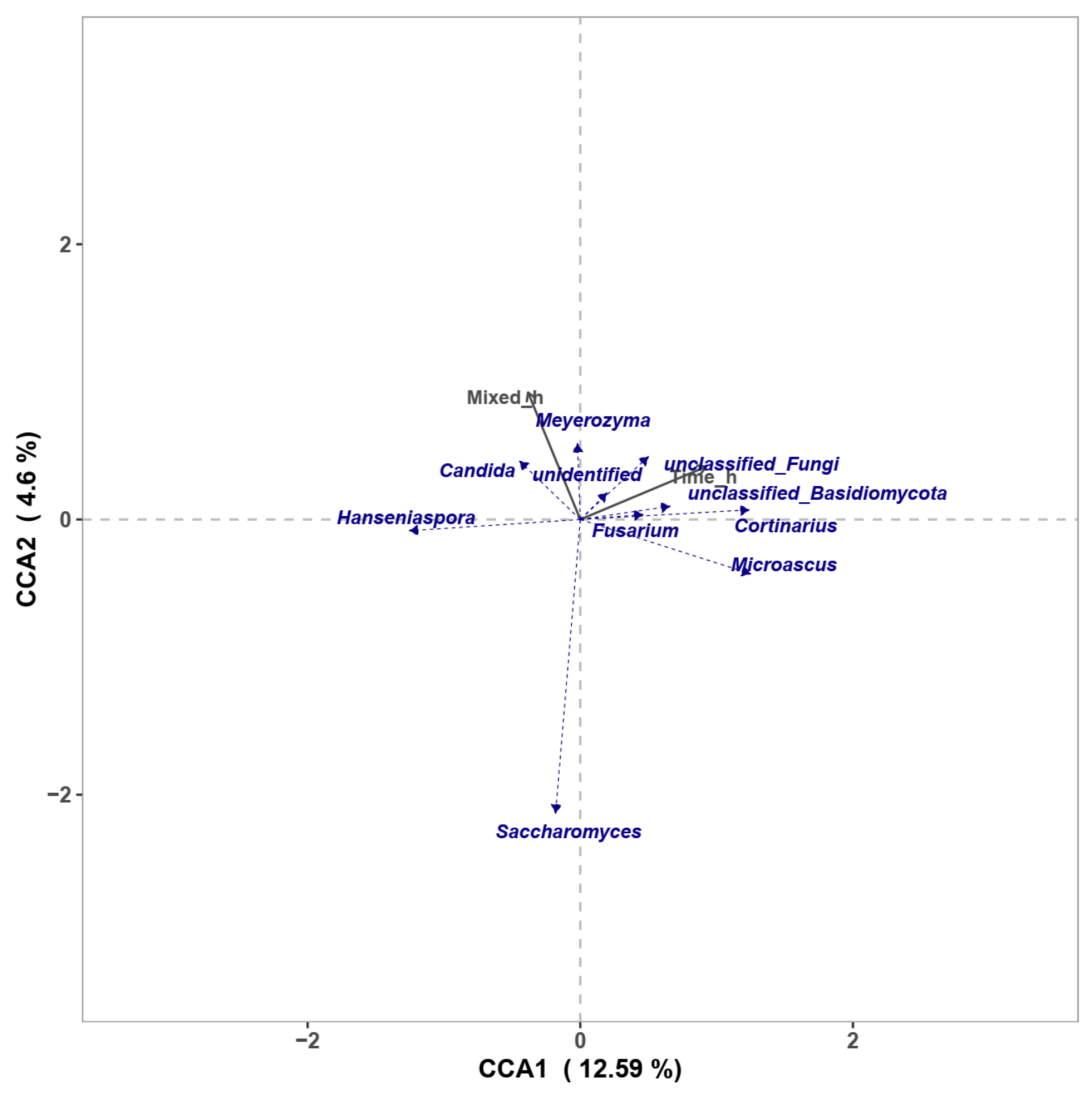
Canonical Correspondence Analysis (CCA) biplot of the total fermentation time and turning interval on the fungal and yeast community structure at the genus level. Time_h: total fermentation time, 0–144 h; Mixed_h: turning interval, 24 h and 48 h.

Canonical correspondence analysis (CCA) was conducted to evaluate the influence of total fermentation time (0 to 144 h) and turning interval (24 h and 48 h) on the fungal and yeast community dynamics (Figure 4). Environmental fitting showed that fermentation time was significantly associated with variation in community composition (R^2^ = 0.594, *p* = 0.006). Turning interval was also significantly associated with the CCA configuration (R^2^ = 0.413, *p* = 0.039). The biplot reveals a clear chronological succession and distinct microbial responses to the aeration strategies. Early-stage yeasts, notably *Saccharomyces* and *Hanseniaspora*, exhibited a strong negative correlation with total fermentation time, indicating their dominance during the initial phases of the process. Furthermore, *Saccharomyces* demonstrated a pronounced negative correlation with the extended 48-h turning interval, suggesting that a more frequent 24-h turning strategy was highly favorable for maintaining its population. In contrast, the 48-h turning interval positively selected for oxidative yeast genera, specifically *Meyerozyma* and *Candida*, which correlated strongly with this less frequent mixing parameter. As fermentation progressed toward 144 h, total elapsed time, rather than the turning interval, primarily drove community structure. This late-stage environment showed a distinct shift toward filamentous fungi and late colonizers, including taxa such as *Microascus* and *Cortinarius*. In addition, given the single fermentation batch per turning treatment and repeated temporal sampling, these relationships should be interpreted as exploratory associations.

In spontaneous cocoa fermentation, multiple factors influence the composition and temporal succession of yeast communities, including post-harvest handling practices, cocoa pod maturity, fermentation system (e.g., wooden-box or heap fermentation), and how often the fermenting bean mass is turned [16,35,36,37]. Yeasts isolated from cocoa fermentations have been identified from diverse geographical regions. Commonly reported genera include *Candida*, *Pichia*, *Saccharomyces*, *Kluyveromyces*, *Hanseniaspora*, *Hansenula*, *Torulaspora*, and *Torulopsis*, with isolates documented in countries such as Brazil, Ghana, Malaysia, Cameroon, Colombia, Cuba, and Ivory Coast [12,36,38,39,40,41,42]. In a previous report, *S. cerevisiae* and *P. kudriavzevii* were the predominant yeast species contributing substantially to cocoa aroma and flavor [43]. *Hanseniaspora* species are frequently detected during the early stages of cocoa fermentation but are progressively replaced by yeasts with greater tolerance to ethanol and elevated temperatures, including *S. cerevisiae*, *P. kudriavzevii*, *P. manshurica*, and *P. kluyveri* [43,44,45,46].

### 3.3. Bacterial Dynamics During Cocoa Fermentation

**Figure 5:**
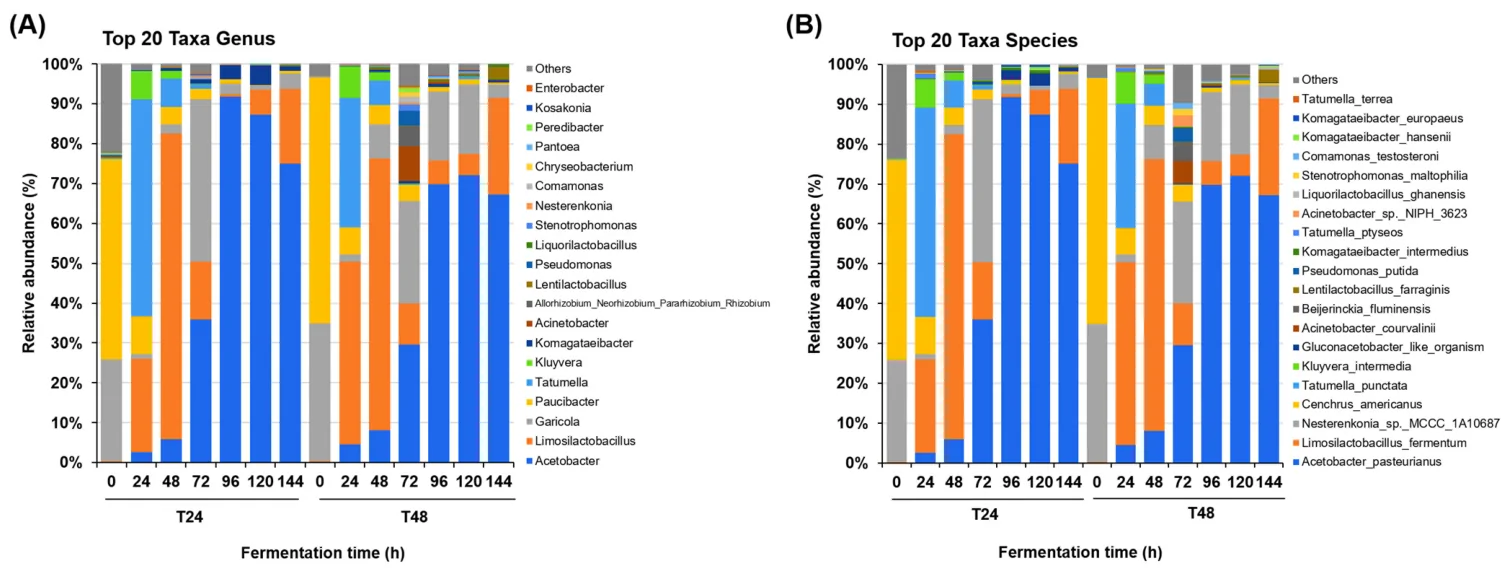
Bacterial population distributions at the genus (**A**) and species (**B**) levels during the cocoa fermentation processes.

Sequencing read processing results and alpha diversity indices for the V3–V4 region are summarized in Tables S3 and S4, respectively. The bacterial growth dynamics during cocoa fermentation for the 20 most abundant bacterial genera and species are presented in Figure 5. In the 24-h turning (T24), *Limosilactobacillus fermentum* attained predominance by 48 h of fermentation, followed by *Acetobacter pasteurianus*, which then dominated (80–90%) from 96 to 144 h of fermentation. More frequent turning in T24 was associated with earlier aeration and a more pronounced succession of AAB, including *Acetobacter* and *Komagataeibacter*. In contrast, the 48-h turning (T48) displayed a lower abundance of *L. fermentum* and *A. pasteurianus*. Prolonging the anaerobic state in the T48 process delayed *Acetobacter* development and increased the relative abundance of *Acinetobacter*. This turning mainly enhanced AAB growth, as indicated by higher cell counts, increased acetic acid concentrations, and higher maximum fermentation temperatures [47]. Our results are consistent with previous reports indicating that *A. pasteurianus* was the dominant AAB during the aerobic phase of spontaneous cocoa bean fermentation [40,46]. Among microaerophilic LAB, citrate-fermenting, acid-tolerant, and ethanol-tolerant strains of *L. plantarum* and *L. fermentum* commonly dominate spontaneous cocoa bean fermentation [48,49]. Moreover, bacterial species-level analysis identified *L. plantarum* and *L. fermentum* as the predominant LAB, whereas *A. pasteurianus*, *A. ghanensis*, and *A. senegalensis* were the principal AAB. These findings indicate the relatively limited species diversity of both LAB and AAB during spontaneous heap fermentation of cocoa beans in Ghana [16,18,47]. As shown in Figure 5, the bacterial population dynamics during turning cocoa fermentation exhibited clear succession patterns influenced by turning frequency, oxygen availability, temperature rise, and metabolite accumulation, consistent with established microbial ecology in cocoa processing [7,8,9,18].

**Figure 6:**
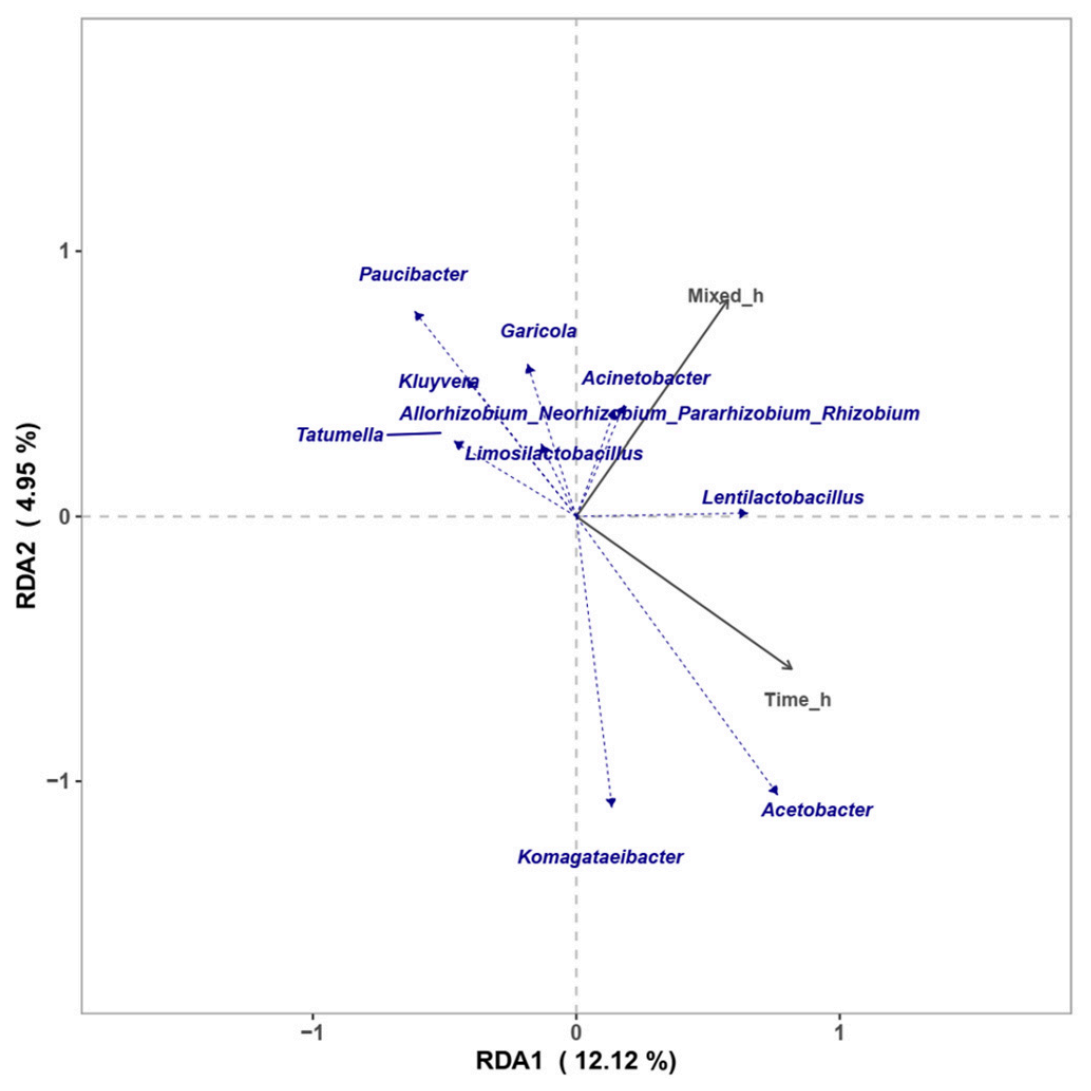
Redundancy Analysis (RDA) biplot of the relationship between bacterial community dynamics (genus level) and fermentation parameters. Time_h: total fermentation time, 0–144 h; Mixed_h: turning interval, 24 h vs. 48 h.

Redundancy analysis (RDA) was performed to evaluate the influence of total fermentation time (0–144 h) and turning interval (24 h vs. 48 h) on bacterial community structure (Figure 6). Environmental fitting showed that fermentation time was significantly associated with variation in community composition (R^2^ = 0.532, *p* = 0.006). Turning interval was also significantly associated with the RDA configuration (R^2^ = 0.716, *p* = 0.002). The biplot (RDA1: 12.12%; RDA2: 4.95%) shows a distinct temporal succession of bacterial taxa and specific responses to the aeration strategies. Early fermentation was dominated by genera such as *Tatumella*, *Paucibacter*, and *Kluyvera*, which showed strong negative correlations with fermentation time, indicating their populations peaked near 0 h and declined as fermentation progressed. As the process approached 144 h, the community shifted toward acid-tolerant organisms, particularly Acetobacter, which showed a strong positive correlation with elapsed time. Moreover, bacterial populations responded differently to the turning intervals. The 24-h turning strategy strongly favored *Komagataeibacter*, as evidenced by its distinct negative correlation with the mixed time interval. This indicates that frequent disruption and increased oxygenation of the fermenting mass promote the proliferation of this specific taxon. Conversely, extending the turning interval to 48 h positively selected for genera such as *Acinetobacter*, *Garicola*, and the *Allorhizobium-Neorhizobium-Pararhizobium-Rhizobium* complex. Differences between turning schedules may partly reflect changes in aeration, although oxygen availability was not measured directly. Future integration of oxygen profiles and physical characterization of the fermentation mass could further elucidate the mechanisms linking turning frequency to fermentation dynamics.

### 3.4. Physicochemical Properties During the Cocoa Fermentation Process

#### 3.4.1. Temperature, Total Acid Content, and pH Value

**Figure 7:**
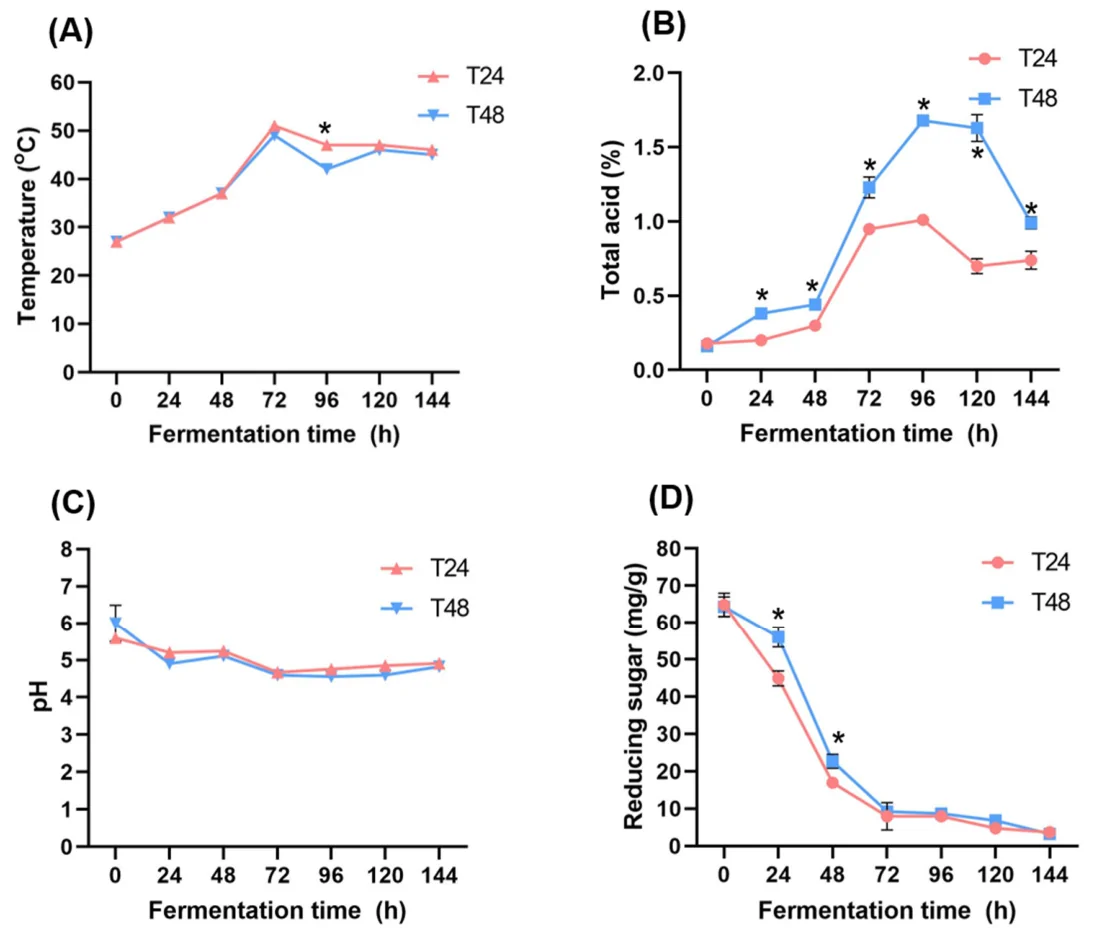
Temperature (**A**), total acid content (**B**), pH (**C**), and reducing sugar (**D**) change during the cocoa fermentation processes. Data are presented as the mean  ±  SD. The asterisk represents a significant difference between T24 and T48 at corresponding time points (*p* < 0.05).

Temperature analysis during cocoa fermentation processes, as depicted in Figure 7A, found that at the beginning of the fermentation process for the T24 process, the temperature began at approximately 28 °C and gradually increased to approximately 50 °C at 72 h due to exothermic microbial activity from yeasts and bacteria, before declining and stabilizing at about 45 °C post-fermentation [32]. The T48 process exhibited temperatures 2–3 °C lower than in the T24, likely due to reduced aeration and slower heat accumulation, consistent with observations that less frequent turning moderated peak temperatures while still supporting flavor development.

The results of the analysis of total acid content and pH during cocoa fermentation are illustrated in Figure 7B,C. The total acid content increased in both fermentation processes. The total acid content during 0–48 h was 0.2–0.5% (*w*/*w*). However, the acid content tended to increase after 48 h. The fermentation T48 process increased acid content to 1.52% (*w*/*w*) at 96 h, then decreased to approximately 1% (*w*/*w*) at 144 h. In contrast, the T24 process showed a maximum increase in acid content of 1% (*w*/*w*) during the 72 h fermentation period and then remained relatively constant. It decreased slightly to 0.75% (*w*/*w*) at the end of the fermentation. These trends reflect intensified accumulation of lactic and acetic acids from LAB and AAB activity, moderated by more frequent aeration in the T24 process. pH measurements in both fermentation processes showed a decreasing trend from an initial pH of 6 to 5, with T24 maintaining a slightly higher pH due to enhanced volatile acid dissipation, consistent with optimal acidification (4.5–5.0) for flavor precursor formation during pulp degradation [50]. Although individual organic acids were not quantified in this study, future time-resolved measurements of lactic and acetic acids using HPLC or enzymatic assays could complement the microbial community profiles and further clarify the metabolic roles of LAB and AAB during fermentation.

The analysis of reducing sugar content during both fermentation processes is shown in Figure 7D. It was found that the reducing sugar content decreased sharply after 24 h of fermentation due to microbial metabolism. In fermentation T24, the reducing sugar content decreased from 64.80 mg/g at 0 h to 7.97 mg/g at 72 h. The residual reducing sugar content in T24 was lower than in T48, which decreased from 64.29 mg/g at 0 h to 9.21 mg/g at 72 h. The reducing sugar content remained constant until the end of fermentation at 3.73 and 3.21 mg/g, respectively. Reducing sugar levels may decline throughout fermentation as they are consumed as energy sources in various biochemical reactions. In addition, enzymatic carbohydrate breakdown releases the corresponding mono-, di-, and oligosaccharides [3,7]. These changes reflect yeast and bacterial consumption of pulp sugars for ethanol and acid production, which are essential flavor precursors [51].

#### 3.4.2. Enzyme Activities

**Figure 8:**
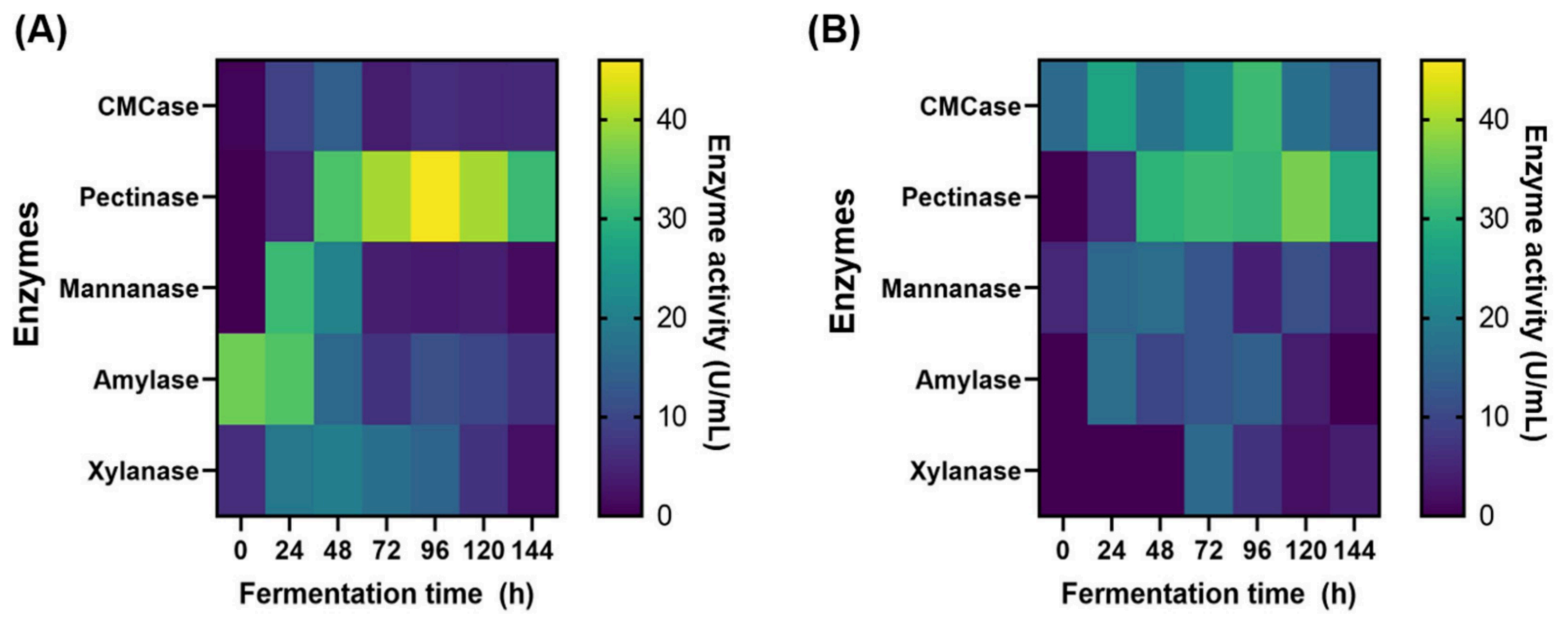
Enzyme activity during the 24 h (**A**) and 48 h (**B**) turning intervals of the cocoa fermentation processes.

During cocoa fermentation, microorganisms including yeast, LAB, and AAB play an important role in cocoa pulp degradation by producing enzymes [13]. The enzyme activities of carboxymethyl cellulase (CMCase), mannanase, pectinase, xylanase, and amylase associated with the cocoa fermentation process were determined using the 3,5-dinitrosalicylic acid (DNS) method (Figure 8). The T24 results, shown in Figure 8A, indicate that CMCase showed very low activity throughout the fermentation period, with relatively constant values. Pectinase showed a rapid initial increase in activity, peaking at 96 h, followed by a steady decrease. Similarly, mannanase activity increased rapidly after 24 h, peaking at 72 h, then declined. Amylase and xylanase peaked at 24 h, but both declined continuously thereafter. In summary, pectinase and mannanase showed high activity during the middle phase of fermentation (48–72 h), while amylase and xylanase showed higher activity early in fermentation, followed by a decline after 48 h. CMCase remained consistently low throughout the process. In the enzyme activity graph for 48-h turning cocoa fermentation (Figure 8B), CMCase showed maximum activity at 48 h, after which it decreased continuously. In contrast, pectinase activity increased gradually, reached a peak at 120 h, and then declined. Mannanase showed maximum activity at 48 h, after which it decreased. Amylase activity was detected between 24 and 96 h, then decreased as fermentation time increased. Xylanase showed low activity throughout, peaking at 72 h and then remaining relatively constant.

The enzyme patterns underscore the coordinated role of hydrolytic enzymes in driving the complex transformation of cocoa pulp and bean components during fermentation. Specifically, the early peak in amylase and xylanase activity corresponds to the initial breakdown of starches and hemicelluloses, which rapidly supplies fermentable sugars supporting the growth of yeasts and lactic acid bacteria [52]. The subsequent mid-phase elevation of pectinase and mannanase activity aligns with the degradation of the mucilaginous pectin and mannans surrounding the beans, facilitating microbial penetration and mass transfer of metabolites such as organic acids and aroma precursors. Several yeast species commonly found during cocoa fermentation show high pectinolytic activity, as demonstrated in vitro and during fermentation. These include *Candida norvegensis*, *C. zeylanoides*, *C. nitrativorans*, *Kluyveromyces fragilis*, *K. marxianus*, *Pichia kudriavzevii*, *P. kluyveri*, and *S. cerevisiae* [43,53,54,55]. The relatively low CMCase activity may indicate a minor but ongoing contribution to cellulose breakdown in cocoa fermentation, potentially helping release additional substrates over time [56]. Importantly, the timing and magnitude of these enzyme activities directly affect the physicochemical environment and the accessibility of flavor precursor molecules, which are crucial for desirable chocolate attributes. Therefore, the observed temporal shifts in enzyme profiles reflect not only microbial succession but also the dynamic conversion of bean macromolecules that underpins flavor maturation and textural improvement in fermented cocoa [4,57].

#### 3.4.3. The Volatile Compounds

**Figure 9:**
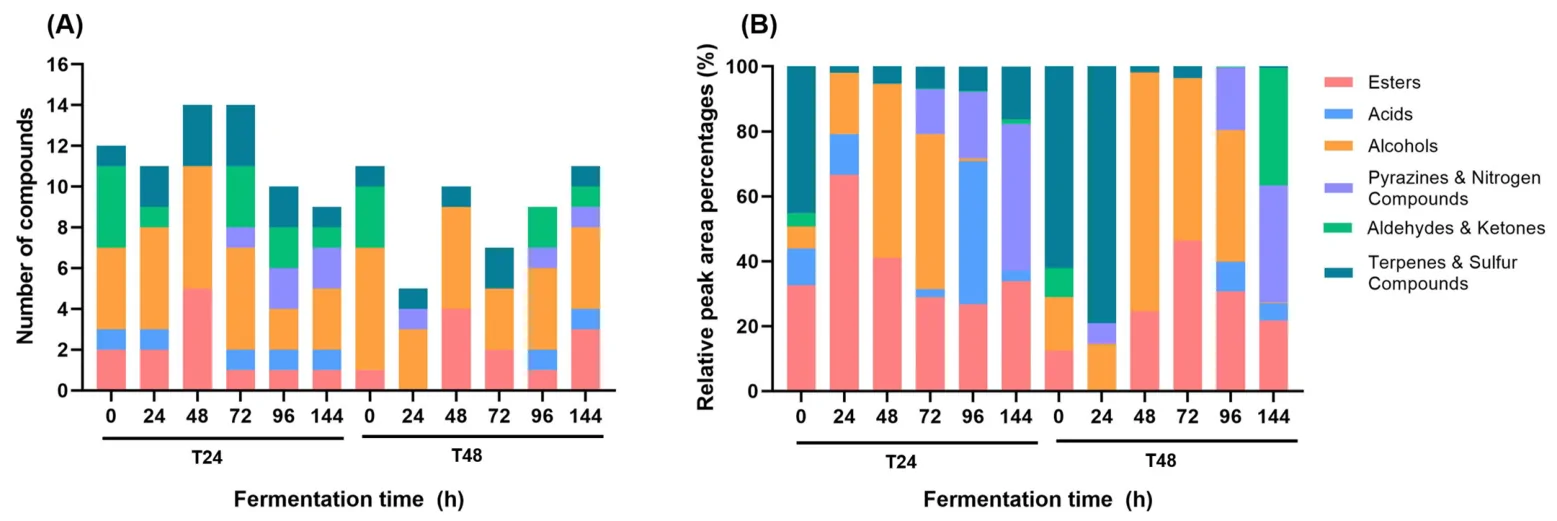
Number of volatile compounds (**A**) and relative peak area percentages of volatile compounds (**B**) during the cocoa fermentation processes.

Chromatographic separation of volatile odor compounds in cocoa bean samples was achieved using the MXT-5 column as the primary analytical column, with the MXT-1701 column used for secondary confirmation. Peaks with an area greater than 500 and a compound-identification confidence score exceeding 80% were retained for subsequent data analysis. The results indicated that 46 volatile compounds were identified, including esters, acids, alcohols, pyrazines and nitrogen compounds, aldehydes and ketones, terpenes, and sulfur compounds, at different fermentation times, as presented in Table S5 and Figure 9. Overall, the number of volatile compounds from T24 methods was higher than from T48 methods. In T24 treatment, compounds associated with fruity aromas, such as ethyl acetate, ethyl butyrate, and isoamyl acetate, were found early in fermentation; meanwhile, nutty and bready aromas, such as 3-Hepten-2-one, 2-methylbutanal, 2,3-dimethylpyrazine, and ethenyl-dimethylpyrazine, were found late in fermentation. Moreover, the number of aldehydes and ketones increased in the T24 process. Traditional on-farm processing of Ecuadorian cocoa also reported increased levels of aldehyde- and ketone-type compounds during the aerobic fermentation step [58]. In the T48 treatment, both fruity and nutty aromas were detected in the final stage of fermentation. Moreover, acid aromas, which are related to off-flavors, were mostly observed in the T24 treatment. The results indicated that the frequency of cocoa bean turning is related to oxygen availability, microbial dynamics, and the volatile profile [59]. Excessive aeration promotes the growth of acetic acid-producing bacteria, resulting in rapid oxidation of ethanol to acetic acid and giving pungent, acidic notes and off-flavors [8,60]. Meanwhile, prolonging the anaerobic phase could promote the ester synthesis from alcohols by yeasts. Under anaerobic conditions, acetyl-CoA accumulated and affected the alcohol acetyltransferase reaction, producing fruity and floral volatile esters [18,61,62]. Moreover, previous studies reported that pyrazine formation was significantly higher with longer turning times, and total pyrazines/trimethylpyrazines were more influenced by aeration time, which later became a source of flavor development during roasting [63]. In addition, the volatile organic compound profiles obtained via the Heracles NEO electronic nose provide semi-quantitative comparative discrimination rather than absolute molar concentrations. Absolute quantification via quantitative HS-SPME-GC-MS with internal standards will be undertaken in future studies to establish exact odor-activity thresholds.

### 3.5. Correlation Analysis Between Microbial Populations and Volatile Compounds

**Figure 10:**
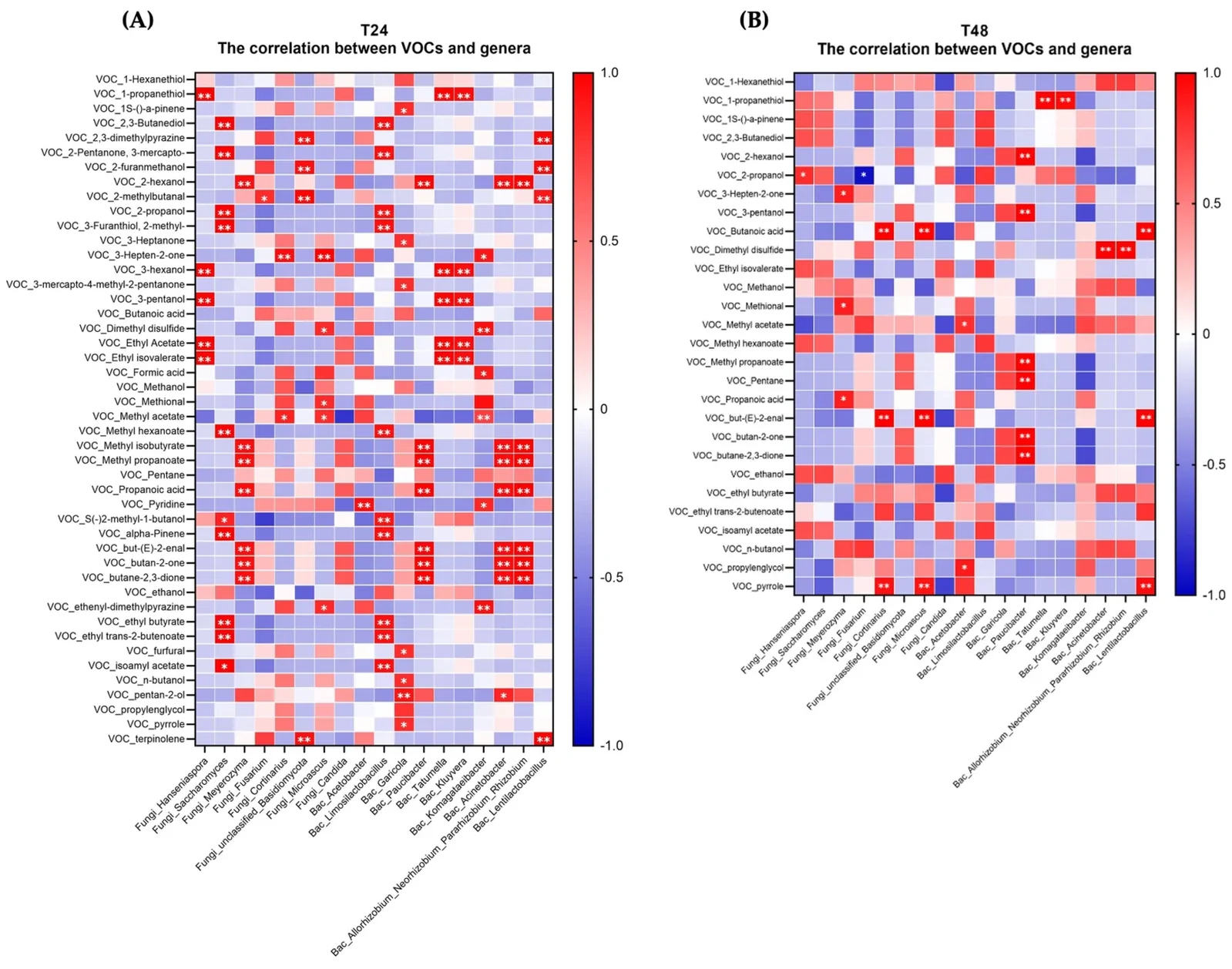
Pearson correlation analysis between key volatile compounds and the most abundant genera of bacteria and fungi during the 24 h (**A**) and 48 h (**B**) turning intervals of the cocoa fermentation processes. * and ** indicate statistically significant correlations at *p* < 0.10 and *p* < 0.05, respectively.

The correlation analysis between the volatile compounds and the relative abundance of bacterial and fungal genera is presented in Figure 10. The results in the T24 treatment revealed that *Saccharomyces*, *Limosilactobacillus*, and *Lentilactobacillus* were significantly positively correlated with key fruity/sweet esters (isoamyl acetate, ethyl butyrate, methyl hexanoate, ethyl trans-2-butenoate), polyols/higher alcohols (2,3-butanediol, 2-propanol, S(-)2-methyl-1-butanol), terpenes (alpha-pinene), and pyrazines (2,3-dimethylpyrazine). Meanwhile, *Lentilactobacillus* and *Acinetobacter* were significantly correlated with butanoic acid and dimethyl disulfide detected in T48 treatments, which were associated with rancid, pungent off-flavor in the final cocoa liquor [64]. *L. fermentum* has been reported to contribute to the production of 3-methylbutanal in cocoa beans. This aldehyde formation was attributed to amino acid degradation during LAB fermentation [65]. Our results were consistent with previous reports showing that *Saccharomyces* correlates directly with higher alcohols and acetate esters, as reported by Díaz-Muñoz and De Vuyst. [43]. Previous studies have shown that *S. cerevisiae* starter cultures can enhance the concentrations of flavor-active esters, aldehydes, ketones, and pyrazines in raw cocoa beans. During cocoa pulp degradation, these strains promote the formation of higher alcohols, which may subsequently be converted into fruity acetate esters through alcohol acetyltransferase activity [45,66]. Because amplicon sequencing provides relative rather than absolute taxon abundances, changes in the relative abundance of a given taxon may partly reflect changes in the abundance of other taxa. Therefore, the observed microbial–volatile correlations should be interpreted as statistical associations across fermentation time points and not as direct causal relationships. In addition, the taxa–volatile correlation analysis was exploratory and based on temporally repeated observations from the same fermentation batch; therefore, temporal dependence and multiple testing may influence the reported associations. Future studies combining independent fermentation replicates, absolute microbial quantification, and targeted metabolite quantification would help validate these relationships.

## 4. Conclusions

In conclusion, turning interval substantially influenced microbial succession, physicochemical changes, and volatile flavor development during cocoa fermentation. LAB and AAB dominated both treatments, while coliform bacteria declined after 72 h and were undetectable by 96 h, indicating effective fermentation and suppression of undesirable microorganisms. *S. cerevisiae* and *H. pseudoguilliermondii* contributed to early ethanol production and pectin degradation, with pectinase representing the predominant enzyme activity. The 24-h turning treatment (T24) was positively associated with fruity and sweet ester compounds through *Saccharomyces*, *Limosilactobacillus*, and *Lentilactobacillus*, whereas the 48-h treatment (T48) showed associations between *Lentilactobacillus* and *Acinetobacter* and volatile markers linked to off-flavor notes. Overall, more frequent turning may promote differences in the microbial community and increase the abundance of volatile compounds previously associated with desirable cocoa aroma attributes. These findings also suggest improvements for industrial fermentation processes and management. However, further studies using multiple independent fermentation boxes across different harvesting seasons are needed to confirm the reproducibility of turning-interval effects and to evaluate their implications for sensory attributes and chocolate product quality.

## Figures and Tables

**Table 1:** Shannon diversity index.

Fermentation Time (h)	Fungal Diversity		Bacterial Diversity	
	T24	T48	T24	T48
0	6.5461	8.0071	3.3004	1.3521
24	3.6222	3.8687	2.6620	2.5858
48	1.5934	3.4164	2.0751	2.3971
72	7.6128	7.6671	2.2935	3.5342
96	6.9457	7.2640	0.6325	1.8099
120	7.2360	6.2622	0.8821	1.5510
144	6.0144	7.5156	1.2701	1.5663

Note: Shannon diversity index values were calculated using a base-2 logarithm (log_2_).

## Data Availability

The raw sequencing data have been deposited in the NCBI Sequence Read Archive (SRA) under BioProject accession number PRJNA1544703. All other relevant data are included within the manuscript and its Supplementary Materials.

## Supplementary Materials

The following supporting information can be downloaded at https://www.mdpi.com/article/10.3390/foods15213814/s1: Table S1: Key possible aroma compounds identified from during the 24 h and 48 h turning intervals of the cocoa fermentation processes; Table S2: Summary on raw data processing of ITS region; Table S3: Summary of alpha diversity indices of ITS region; Table S4: Summary on raw data processing of V3-V4 region; Table S5: Summary of alpha diversity indices of V3-V4 region.
