# Supplementary material for: Influence of Turning Intervals on Microbial Succession and Volatile Aroma Development During On-Farm Cocoa Bean Fermentation
Source: Foods. 2026 Oct 25;15(21):3814. doi: 10.3390/foods15213814 (PMC13762886; doi:10.3390/foods15213814)
Supplement: Supplementary file 1 [file foods-15-03814-s001.zip › foods-4615669-supplementary.pdf]

Supplementary Material

Table S1. Key possible aroma compounds identified from during the 24 h and 48 h turning intervals of the cocoa fermentation processes.

No.	Possible compounds	Order description	RT (s)	T24 treatment						T48 treatment					
				0 h	24 h	48 h	72 h	96 h	144 h	0 h	24 h	48 h	72 h	96 h	144 h
1	1-Hexanethiol	Burnt, Fatty, Garlic	49.32	nd	923.98	nd	2319.75	nd	nd	nd	nd	nd	1659.86	nd	1191.56
2	1-propanethiol	Alliaceous, Cabbage, Onion	24.78	nd	1618.16	nd	nd	nd	nd	nd	85691.21	nd	nd	nd	nd
3	1S-()-a-pinene	Fresh, Herbaceous, Pine	57.1	nd	nd	nd	6343.50	nd	nd	nd	nd	862.11	nd	nd	nd
4	2,3-Butanediol	Creamy, Fruity, Odorles	42.62	nd	nd	574.82	nd	nd	nd	nd	nd	524.9	nd	nd	nd
5	2,3-dimethylpyrazine	Baked, Bread, Burnt sugar	56.67	nd	nd	nd	nd	nd	2839.52	nd	nd	nd	nd	nd	nd
6	2-furanmethanol	Alcoholic, Bread, Burnt sugar	49.21	nd	nd	nd	nd	nd	689.70	nd	nd	nd	nd	nd	nd
7	2-hexanol	Cauliflower, Chemical, Fatty	44.31	976.59	nd	nd	nd	nd	nd	1215.79	nd	nd	nd	nd	nd
8	2-methylbutanal	Almond, Apple, Burnt	29.29	4200.75	530.44	nd	nd	nd	13558.29	nd	nd	nd	nd	nd	nd
9	2-Pentanone, 3-mercapto-	Horseradish, Meaty, Metallic	54.25	nd	nd	1000.14	nd	nd	nd	nd	nd	nd	nd	nd	nd
10	2-propanol	Acetone, Alcoholic, Ethanol	18.39	nd	nd	20669.91	nd	nd	nd	14471.76	21261.69	25299.89	nd	nd	11058.66
11	3-Furanthiol, 2-methyl	Barbeque, Broth, Fatty	49.27	nd	nd	1854.09	nd	nd	nd	nd	nd	nd	nd	nd	nd
12	3-Heptanone	Cinnamon, Fatty, Fruity	53.32	nd	nd	nd	2385.75	nd	nd	nd	nd	nd	nd	nd	nd
13	3-Hepten-2-one	Caraway, Grassy, Green	56.00	nd	nd	nd	1856.55	4034.72	nd	nd	nd	nd	nd	2322.80	nd
14	3-hexanol	Alcoholic, Etheral, Medicinal	43.62	nd	nd	nd	nd	nd	nd	nd	nd	nd	nd	nd	nd
15	3-mercapto-4-methyl-2-pentanone	Blackcurrant	52.00	nd	nd	nd	3396.70	nd	nd	nd	nd	nd	nd	nd	nd
16	3-pentanol	Fruity, Green, Nutty	32.72	nd	14515.72	nd	nd	nd	nd	12598.48	nd	nd	nd	nd	nd
17	Alpha-Pinene	Camphor, Citrus, Earthy	57.05	nd	nd	700.8	nd	nd	nd	nd	nd	nd	nd	nd	nd
18	But-(E)-2-enal	Floral, Green, Plastic	28.35	2809.65	nd	nd	nd	nd	nd	7805.41	nd	nd	nd	nd	1046487.00
19	Butan-2-one	Acetone, Butter, Cheese	23.69	814.82	nd	nd	nd	nd	nd	1167.68	nd	nd	nd	nd	nd
20	Butane-2,3-dione	Butter, Caramelized, Chlorine	22.89	1498.35	nd	nd	nd	nd	nd	2249.30	nd	nd	nd	nd	nd
21	Butanoic acid	Butter, Cheese, Penetrating	45.77	nd	nd	nd	1949.53	nd	1220.14	nd	nd	nd	nd	nd	5631.65
22	Dimethyl disulfide	Cabbage, Cheese (ripened), Garlic	37.74	nd	nd	nd	nd	3651.6	nd	nd	nd	nd	3240.61	nd	nd
23	Ethanol	Alcoholic, Ethanol, Etheral	17.24	82348.01	1017597.00	1487418.00	1470400.00	115343.10	49912.26	85656.96	670950.60	1436499.00	678865.50	767554.50	62438.54

No.	Possible compounds	Order description	RT (s)	T24 treatment						T48 treatment					
				0 h	24 h	48 h	72 h	96 h	144 h	0 h	24 h	48 h	72 h	96 h	144 h
24	Ethenyl-dimethylpyrazine	Earthy, Musty	68.68	nd	nd	nd	nd	1954.88	nd	nd	nd	nd	nd	nd	nd
25	Ethyl acetate	Acidic, Butter, Caramelized	24.73	nd	69609.01	nd	nd	nd	nd	nd	nd	nd	nd	nd	nd
26	Ethyl butyrate	Acetone, Banana, Bubblegum	43.49	nd	nd	2624.41	nd	nd	nd	nd	nd	nd	8223.86	nd	6622.35
27	Ethyl isovalerate	Anise, Apple, Blackcurrant	49.28	nd	509.47	nd	nd	nd	nd	nd	nd	2558.06	nd	nd	nd
28	Ethyl trans-2-butenoate	Alliaceous, Chemical, Pungent	45.87	nd	nd	1773.47	nd	nd	nd	nd	nd	2595.35	nd	nd	3543.58
29	Formic acid	Acidic, Pungent, Vinegar	22.94	nd	7334.98	nd	nd	38945.38	nd	nd	nd	nd	nd	nd	nd
30	Furfural	Almond, Baked, Benzaldehyde	45.98	nd	nd	nd	2574.33	nd	nd	nd	nd	nd	nd	nd	nd
31	Isoamyl acetate	Apple, Banana, Ester	51.93	nd	nd	694.13	nd	nd	nd	nd	nd	3815.93	nd	nd	nd
32	Methanol	Alcoholic, Pungent, Strong	15.95	6848.24	9351.59	8697.54	13000.75	9247.60	6684.79	6305.66	8620.22	9293.71	11366.04	9646.25	6012.12
33	Methional	Baked potato, Creamy, Earthy	54.78	nd	nd	nd	nd	3974.96	nd	nd	nd	nd	nd	2953.87	nd
34	Methyl acetate	Blackcurrant, Etheral, Fragrant	19.85	nd	nd	16849.39	42438.56	42409.28	22978.14	nd	nd	nd	43970.47	39862.04	32085.60
35	Methyl hexanoate	Acetone, Fresh, Fruity	55.99	nd	nd	539.33	nd	nd	nd	nd	nd	569.2	nd	nd	nd
36	Methyl isobutyrate	Apple, Floral, Fruity	31.55	4100.32	nd	nd	nd	nd	nd	nd	nd	nd	nd	nd	nd
37	Methyl propanoate	Apple, Etheral, Fresh	25.93	750.98	nd	nd	nd	nd	nd	1051.85	nd	nd	nd	nd	nd
38	n-Butanol	Alcoholic, Amyl alcohol, Banana	28.82	nd	nd	nd	2042317.00	nd	nd	6639.70	nd	nd	2176944.00	1899654.00	nd
39	Pentan-2-ol	Alcoholic, Etheral, Fermented	32.69	17179.77	nd	nd	15190.74	nd	nd	nd	nd	nd	nd	nd	nd
40	Pentane	Alkane, Gasoline, Pleasant	19.79	8215.65	nd	nd	nd	10917.61	nd	6420.78	nd	nd	nd	nd	nd
41	Propanoic acid	Acidic, Beefy, Cheese	53.29	941.42	nd	nd	nd	nd	nd	nd	nd	nd	nd	6634.28	nd
42	Propylenglycol	Alcoholic, Caramelized, Odorless	36.01	nd	nd	582.19	4338.88	nd	nd	nd	nd	1731.41	nd	5720.83	4550.63
43	Pyridine	Amine, Burnt, Cold meat fat	39.08	nd	nd	nd	nd	6019.49	4693.37	nd	nd	nd	nd	nd	nd
44	Pyrrole	Chloroform, Coffee, Cracker	39.58	nd	nd	nd	4942.47	nd	nd	nd	1359.96	nd	nd	6161.77	17206.86
45	S(-)2-methyl-1-butanol	Fresh, Fruity, Malty	37.14	nd	1410.41	2653.08	nd	nd	nd	nd	nd	nd	nd	nd	nd
46	Terpinolene	Anisic (slightly), Citrus, Fresh, Fruity,	68.75	nd	nd	nd	nd	nd	557.92	nd	nd	nd	nd	nd	nd

nd = not detected.

Table S2. Summary on raw data processing of ITS region

Treatment	Fermentation time	Raw Reads	Clean Reads	Denoised Reads	Merged Reads	Non-chimeric Reads
T24	0 h	159814	141031	140966	140321	135878
T24	24 h	160010	145239	145186	144714	139615
T24	48 h	160108	142713	142659	124122	123598
T24	72 h	159953	135864	135636	128985	127749
T24	96 h	159976	139034	138837	122102	121275
T24	122 h	159963	136790	136630	101943	101151
T24	144 h	160285	136986	136983	46147	45246
T48	0 h	159941	143666	143450	143253	141735
T48	24 h	159794	145624	145537	134661	132166
T48	48 h	159987	144317	144265	142964	136251
T48	72 h	159920	136917	136797	97613	96713
T48	96 h	160039	140212	140201	133098	131810
T48	122 h	159788	141923	141844	134263	131882
T48	144 h	160175	137994	137748	129220	127940

Table S3. Summary of alpha diversity indices of ITS region

Treatment	Fermentation time	Feature	ACE	Chao1	Simpson	Shannon	PD_whole_tree	Coverage
T24	0 h	378	378.2937	378	0.9662	6.5461	57.1362	1
T24	24 h	203	203.6986	203.3333	0.8084	3.6222	13.8176	1
T24	48 h	119	121.3424	120.2	0.4168	1.5934	10.3444	1
T24	72 h	945	945.8913	945.1429	0.9783	7.6128	53.983	1

T24	96 h	1415	1415.9152	1415.1852	0.9572	6.9457	63.2211	1
T24	122 h	1100	1100.415	1100.0345	0.9722	7.236	63.1165	1
T24	144 h	115	115	115	0.9804	6.0144	14.7226	1
T48	0 h	570	570	570	0.9939	8.0071	56.8574	1
T48	24 h	162	162.3051	162	0.8416	3.8687	8.5077	1
T48	48 h	153	153.7466	153.375	0.7999	3.4164	24.5676	1
T48	72 h	508	508.182	508	0.9917	7.6671	38.4574	1
T48	96 h	511	511.2288	511	0.9784	7.264	34.8696	1
T48	122 h	946	948.3971	946.5844	0.9495	6.2622	56.3606	0.9999
T48	144 h	1109	1109.7894	1109.1667	0.9694	7.5156	58.7444	1

Table S4. Summary on raw data processing of V3-V4 region

Treatment	Fermentation time	Raw Reads	Clean Reads	Denoised Reads	Merged Reads	Non-chimeric Reads
T24	0 h	160002	147554	147512	147408	147340
T24	24 h	101168	93369	93306	93084	89061
T24	48 h	103164	94313	94298	94158	93050
T24	72 h	152526	142124	142120	142067	141944
T24	96 h	119954	110666	110657	110435	108909
T24	122 h	128730	118317	118293	118066	112589
T24	144 h	133930	123731	123660	123504	122410
T48	0 h	107116	98386	98370	98354	98302
T48	24 h	159517	148349	148282	147892	142936

T48	48 h	156001	143442	143412	143293	142314
T48	72 h	159980	147624	147607	147470	147183
T48	96 h	159717	147232	147215	147041	146892
T48	122 h	160169	148880	148870	148775	148606
T48	144 h	132560	121568	121530	121383	118303

Table S5. Summary of alpha diversity indices of V3-V4 region

Treatment	Fermentation time	Feature	ACE	Chao1	Simpson	Shannon	PD_whole_tree	Coverage
T24	0 h	241	241.2823	241	0.6817	3.3004	28.1924	1
T24	24 h	90	90.7989	90.0435	0.7187	2.662	5.2018	1
T24	48 h	139	139.4807	139.05	0.5812	2.0751	18.592	1
T24	72 h	142	142	142	0.6888	2.2935	10.3933	1
T24	96 h	62	62.7344	62.0588	0.1557	0.6325	10.6514	1
T24	122 h	57	57.3124	57	0.2342	0.8821	9.8976	1
T24	144 h	70	70	70	0.4084	1.2701	4.0265	1
T48	0 h	83	83	83	0.5006	1.3521	8.8378	1
T48	24 h	86	86	86	0.7467	2.5858	5.6935	1
T48	48 h	87	87	87	0.6596	2.3971	5.328	1
T48	72 h	139	139.3663	139	0.8293	3.5342	10.6716	1
T48	96 h	188	188	188	0.4837	1.8099	12.4405	1
T48	122 h	105	105.4511	105	0.4527	1.551	8.5611	1
T48	144 h	59	59.3651	59	0.5027	1.5663	4.6376	1
